# Reply to “The Carotid Sinus as a Viscometer”

**DOI:** 10.3390/diagnostics10110925

**Published:** 2020-11-10

**Authors:** Ui Yun Lee, Chul In Kim, Gyung Ho Chung, Jinmu Jung, Hyo Sung Kwak

**Affiliations:** 1 Division of Mechanical Design Engineering, Jeonbuk National University, Jeonju 54896, Korea; euiyun93@naver.com (U.Y.L.); kci2850@jbnu.ac.kr (C.I.K.); 2 Department of Radiology and Research Institute of Clinical Medicine of Jeonbuk National University, Biomedical Research Institute of Jeonbuk National University Hospital, Jeonju 54907, Korea; chunggh@jbnu.ac.kr; 3 Hemorheology Research Institute, Jeonbuk National University, Jeonju 54896, Korea

Above all, we would like to express our sincere thanks and appreciation for writing your comment on our research. It is such a great honor to have so much interest in our work from so many researchers. We are glad to have an opportunity to write a reply to “The Carotid Sinus as a Viscometer”. We are very interested in the hypothesis you have mentioned. As you mentioned, from a hemorheological point of view, the carotid sinus is a unique region in which rheological factors of blood changes dynamically compared to the other locations, due to recirculation. Blood has the property of a non-Newtonian fluid whose viscosity varies according to different shear rates, defined as velocity gradient to vessel diameter. Diverse vessel diameters and blood flow velocities can be found within the human body, which means that blood flows in a wide range of shear rates from low to high in the vessels. Your hypothesis that the sinus area performs as a transducer of blood viscosity might be reasonable. The recirculation flow generated in the carotid sinus, a bulb-shaped physical anatomy, might play a role in mimicking a wide range of shear rates similar to the blood flow conditions in the human body. As the recirculation occurs, the large variation in shear rates from low to high might be found, and accordingly, non-Newtonian viscosity changes might be monitored. Additionally, this leads to variations in oscillatory shear index. Thus, we agree with your hypothesis that the sinus region might function as a real-time sensor to detect the dynamic rheological changes of blood.

